# Very large fluxes of methane measured above Bolivian seasonal wetlands

**DOI:** 10.1073/pnas.2206345119

**Published:** 2022-08-01

**Authors:** James L. France, Mark F. Lunt, Marcos Andrade, Isabel Moreno, Anita L. Ganesan, Thomas Lachlan-Cope, Rebecca E. Fisher, David Lowry, Robert J. Parker, Euan G. Nisbet, Anna E. Jones

**Affiliations:** ^a^British Antarctic Survey, Natural Environment Research Council, Cambridge, CB3 0ET, United Kingdom;; ^b^Department of Earth Sciences, Royal Holloway University of London, Egham, TW20 0EX, United Kingdom;; ^c^Environmental Defense Fund, London, EC3M 1DT, United Kingdom;; ^d^School of GeoSciences, University of Edinburgh, Edinburgh, EH9 3FF, United Kingdom;; ^e^Laboratory for Atmospheric Physics, Institute for Physics Research, Universidad Mayor de San Andres, 8635 La Paz, Bolivia;; ^f^Department of Atmospheric and Oceanic Sciences, University of Maryland, College Park, MD 20742;; ^g^School of Geographical Sciences, University of Bristol, Bristol, BS8 1SS, United Kingdom;; ^h^National Centre for Earth Observation, University of Leicester, Leicester, LE4 5SP, United Kingdom

**Keywords:** methane, climate, wetlands, greenhouse gases

## Abstract

Methane (CH_4_) mole fractions from the large semiseasonal Llanos de Moxos wetlands (∼70,000 km^2^) in northern Bolivia were measured by aircraft flights and ground sampling during early March 2019 (late wet season). Daily fluxes of CH_4_ determined from the measurements using box models and inverse modeling were between 168 (± 50) and 456 (± 145) mg CH_4_⋅m^−2^⋅d^−1^ for the areas overflown, very high compared with those of previous Amazon basin studies. If the seasonality of the CH_4_ emissions is comparable to other parts of the Amazon Basin, the region could contribute as much as 8% of annual Amazonian CH_4_ emissions.

Here, we present results from an aircraft and ground-based campaign to measure methane (CH_4_) emissions from the seasonal Llanos de Moxos and Rio Mamoré floodplain wetlands of northeastern Bolivia. This was a wide-area-scale airborne measurement of CH_4_ emissions from these major wetlands in the Bolivian component of the Amazon Basin. Previous studies focusing on the Brazilian Amazon Basin found large CH_4_ fluxes, averaging 27 mg CH_4_⋅m^−2^⋅d^−1^ (maximum ∼80 CH_4_⋅m^−2^⋅d^−1^) (1), while work using multiyear aerial measurements found an average of 17.4 ± 3.9 mg CH_4_⋅m^−2^⋅d^−1^ from the Amazonian region as a whole (2). Although the importance of the Bolivian “*sabana inundable*” (flood-prone savannah) as a major CH_4_ source has been inferred from large underestimates of CH_4_ emissions by land-surface models compared with satellite data (3), there have been no previous aircraft studies in this part of Amazonia to test for large-scale emissions to the atmosphere.

The Llanos de Moxos are extensive (∼70,000 km^2^) seasonal wetlands in northeastern Bolivia, flanking the Rio Mamoré at a latitude of about 12 to 15°S with an outer-tropical wet season from December through March. We measured atmospheric CH_4_ mole fractions during aircraft sorties on 2 days in March 2019, aiming to determine bulk wetland-scale CH_4_ fluxes. Early March is in the region’s late wet season, with temperatures still warm and water levels high from the cumulative rainfall. Air samples were captured during flights and in a parallel campaign at ground level adjacent to the wetlands. These air samples were analyzed for δ^13^C_CH4_ and their isotopic source signatures used to indicate the pathway of CH_4_ formation and thus whether the source is likely to be from wetland (4).

Globally averaged atmospheric CH_4_ mole fractions have risen persistently since 2007, with further acceleration from 2014 and record growth in 2020 (5). Concurrently, atmospheric δ^13^C_CH4_ has become isotopically lighter since 2007 in all latitude bands, most plausibly explained by increasing emissions from biogenic sources such as wetlands and cattle (6). Large-scale tropical wetlands are major sources of CH_4_ emissions, both in South America (1, 2) and Africa (7). Saunois et al. (8) estimated that wetlands account for ∼30% of the total CH_4_ flux to the atmosphere, but those wetlands with the greatest emissions (in the tropics) are the least well-characterized, and climate change feedbacks, especially in southern Amazonia, are poorly understood (9). Methane emissions from tropical wetlands respond to increasing temperature and precipitation, both key factors in interannual variation. The warming may be feeding warming, driving sustained growth in the global CH_4_ burden (5).

## Results

**Fig. 1.**
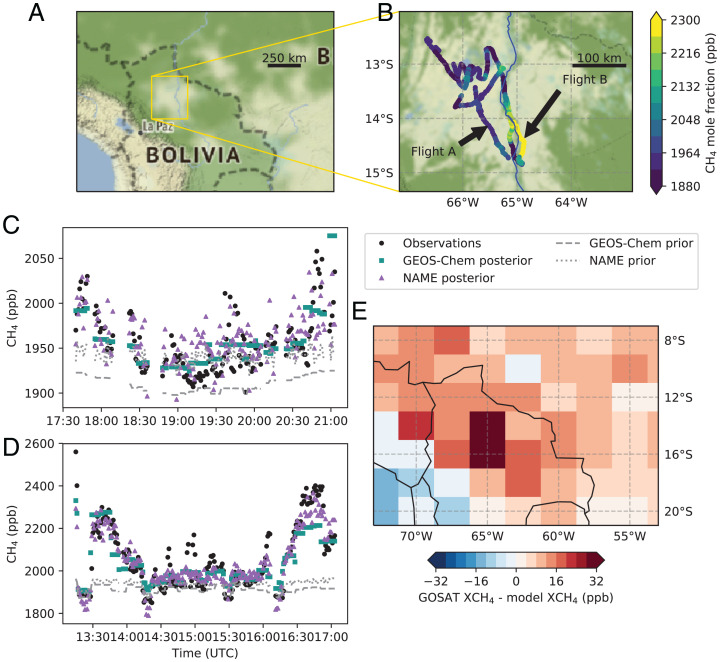
(*A*) Location of campaign, flights starting and ending at Trinidad, Bolivia. (*B*) Flight tracks and observed atmospheric methane mixing ratios. (*C*) Comparison between observations of methane mixing ratios with model prior and posterior derived methane mixing ratios for Flight A, 8 March 2019. Gaps in the data are due to in-flight calibrations and profile climbs outside of the boundary layer. (*D*) As *C*, for Flight B, 9 March 2019. Flight A is primarily over the seasonal wetland to the northwest of Trinidad, and Flight B follows the main river channel (Rio Mamoré) to ∼13°S and then resamples regions from Flight A. Note the different *y* axis scales in *C* and *D*. Wind direction was primarily from the northwest for Flight A and from the north for Flight B. (*E*) Mean excess methane measured from GOSAT satellite data compared with simulated methane from the GEOS-Chem atmospheric chemistry transport model over the period from January to March from 2010 to 2020.

Two flights were conducted with both continuous measurement and bag sampling on board. Flight A was in the late afternoon (8 March 2019), with a planetary boundary layer (PBL) thickness of at least 1,400 m; Flight B was a morning flight (9 March 2019) with an average PBL thickness of 620 m. The PBL depths are estimated from vertical profile measurements of air temperature during flights. Flight tracks, CH_4_ atmospheric mixing ratios, and locations are shown in Fig. 1 *A* and *B*.

Maximum CH_4_ enhancement above background mixing ratios was 200 ppb on Flight A and 550 ppb Flight B. Using Keeling plot analysis on sampled air, the overall δ^13^C_CH4_ source signature of CH_4_ enhancements during Flight A was determined to be −56.1 ± 2.9‰ and on Flight B was −58.6 ±4.1‰ (95% confidence), consistent with the results of the parallel ground-based downwind sampling campaign which gave a δ^13^C_CH4_ source signature of −55.5 ± 4.0‰.

Methane fluxes from the aerial surveys of the Llanos de Moxos were calculated using three methods.

1) Using a simple boundary layer box-model mass balance approach (10) assuming that the wetland surface emits at a constant rate per square kilometer, giving a flux of 168 (± 50) mg CH_4_⋅m^−2^⋅d^−1^ and 456 (± 145) mg CH_4_⋅m^−2^⋅d^−1^ for the areas overflown by Flights A and B, respectively.
2) Using a Bayesian inversion of measurement data and a nested version of the GEOS-Chem model, an average flux of 384 (± 48) mg CH_4_⋅m^−2^⋅d^−1^ was determined along the path of the main Rio Mamoré river channel corresponding to the flight path of Flight B (Fig. 1).
3) Using a hierarchical Bayesian inversion with the high-resolution Numerical Atmospheric dispersion Modeling Environment (NAME) regional Lagrangian transport model (11), an average flux of 156 (± 48) mg CH_4_⋅m^−2^⋅d^−1^ over the same Flight B main river channel.

To establish whether the measured CH_4_ enhancements are a regular regional feature during the wet season, we compared GOSAT (Greenhouse gases Observing SATellite) satellite retrievals over the Llanos de Moxos with mole fractions simulated by the GEOS-Chem model over the period from January to March from 2010 to 2020 (Fig. 1*E* and *SI Appendix*, *Supplementary Materials and Methods*). To scale the model column enhancements to the GOSAT-observed data, average required CH_4_ flux from the Llanos de Moxos wetland would be ∼210 (± 55) mg CH_4_⋅m^−2^⋅d^−1^. For 2019 only, the required flux was ∼355 (± 115) mg⋅m^−2^⋅d^−1^, consistent with the fluxes determined from Flight B (although with high uncertainty).

## Discussion

CH_4_ enhancements observed in flight were very large (>500 ppb for Flight B) and prolonged through the measurement period. The variability in the measured atmospheric mixing ratios of CH_4_ is a function of the depth of the PBL and of the emission flux of methane from the wetland below. These measurements are well-simulated by the models, with close correspondence of box-model and three-dimensional modeling results. As the flights were predominantly over the main river channel, there are larger uncertainties for fluxes over the wider Llanos de Moxos. These CH_4_ fluxes are greater than found in other Amazonian region-scale measurements, with the largest comparable previously determined flux of ∼80 mg CH_4_⋅m^−2^⋅d^−1^ for eastern Amazonia in mid-February (1). We note, however, that early March would be around the expected late rainy seasonal peak of emission. The results of our comparison of modeled wetland emissions with satellite enhancements over the Llanos de Moxos are similar to those of Parker et al. (3). They found wetland modeling underestimated fluxes from a broader region including northern Bolivia, particularly during peak inundation and flux (January to March).

The isotopic results are closely comparable to results from parallel southern outer tropical wetlands in Zambia (12) and to previously reported overflight measurements from Brazilian Amazonian wetlands that gave a δ^13^C_CH4_ source signature of −58.8‰ (13). Such sources, if increasing, would help drive the global methane burden to more negative δ^13^C_CH4_ emissions. Our measurement observations hint at two modes of emission from the region: a seasonally inundated wetland flux from the plains (Flight A, Fig. 1*C*) and a more intense flux centered on tree-lined permanently inundated area along the major river channel (Flight B, Fig. 1*D*). This interpretation is supportive of findings of large CH_4_ fluxes across a number of rivers in the central Amazon Basin where they calculated that tree-mediated fluxes were equal to, if not greater than, all other combined ground-level fluxes (14).

In the 11-year GOSAT satellite record, consistent large (albeit coarse-resolution) enhancements over the Llanos de Moxos recorded add confidence that the observed CH_4_ enhancement is not a one-off result (Fig. 1*E*). Assuming that the fluxes from the Llanos de Moxos region have a methane flux seasonality comparable to the Rio Branco site (67.9°W, 9.3°S) (2), scaling up the observations from Flight A (as this flight is the most representative of the region as a whole) to annualized emissions yields rates of ∼3.6 Tg CH_4_/y for the whole Llanos de Moxos wetland region. This flux would be equivalent to ∼8% of the estimated total Amazonian CH_4_ emission (2), showing that the northern Bolivian wetlands are likely to be a significant contributor to global emissions. This is a preliminary result from a brief campaign of opportunity and further study is required to assess the seasonality of emissions and the possibility of disproportionately large emissions from riverine forests and wetlands.

## Materials and Methods

Measurements were made from the BAS twin-otter aircraft using a Los Gatos Research uGGA instrument calibrated in-flight. Spot samples were collected in Tedlar bags in-flight over wetlands and at ground level downwind from wetlands and returned to the United Kingdom for analysis. Fluxes of CH_4_ were determined from the measurements three ways, using simple box-model mass-balance methods (10) and inverse modeling using the GEOS-Chem (7) and NAME models (15). GOSAT satellite column XCH_4_ data from 2010 to 2020 were compared with XCH_4_ enhancements simulated by GEOS-Chem over the wetlands to determine whether CH_4_ enhancements seen during this campaign were consistent with previous wet seasons. For further details see *SI Appendix*, *Supplementary Materials and Methods*.

## Supplementary Material

Supplementary File

## Data Availability

All original data within this manuscript have been deposited in the publicly accessible Mendeley Data repository (doi: 10.17632/pm4sw7hy3b.1).
