# Supplementary material for: Very large fluxes of methane measured above Bolivian seasonal wetlands
Source: Proc Natl Acad Sci U S A. 2022 Aug 1;119(32):e2206345119. doi: 10.1073/pnas.2206345119 (PMC9371645; doi:10.1073/pnas.2206345119)
Supplement: Supplementary File [file pnas.2206345119.sapp.pdf]

Supplementary Information for

Very Large Fluxes of Methane Measured Above Bolivian Seasonal Wetlands.

James L. France[a,b,c,1], Mark F. Lunt[d], Marcos Andrade[e,f], Isabel Moreno[e], Anita L. Ganesan[g], Thomas Lachlan-Cope[a], Rebecca E. Fisher[b], David Lowry[b], Robert J. Parker[h], Euan G. Nisbet[b], and Anna E. Jones[a]

[a]British Antarctic Survey, Natural Environment Research Council, Cambridge, CB3 0ET, United Kingdom;

[b]Department of Earth Sciences, Royal Holloway University of London, Egham, TW20 0EX, United Kingdom;

[c]Environmental Defense Fund, London, EC3M 1DT, United Kingdom;

[d]School of GeoSciences, University of Edinburgh, Edinburgh, EH9 3FF, United Kingdom;

[e]Laboratory for Atmospheric Physics, Institute for Physics Research, Universidad Mayor de San Andres, 8635 La Paz, Bolivia;

[f]Department of Atmospheric and Oceanic Sciences, University of Maryland, College Park, MD 20742;

[g]School of Geographical Sciences, University of Bristol, Bristol, BS8 1SS, United Kingdom; and

[h]National Centre for Earth Observation, University of Leicester, Leicester, LE4 5SP, United Kingdom

Correspondence to: James L France

Email: james.france@rhul.ac.uk

This PDF file includes:

Supplementary text

SI References

Supplementary Materials and Methods

The aircraft used was the British Antarctic Survey's Twin-Otter VP-FAZ (https://tinyurl.com/y2afydkj). On board was a Los Gatos Research uGGA (ultraportable Greenhouse Gas Analyzer) for measuring in-situ atmospheric methane mole fraction (at 1Hz and ~1ppb precision) and an open sample line to allow spot sample tedlar bag filling. The uGGA was connected to a 3-gas manual calibration deck to allow in-flight calibration and performed approximately every hour of flight (1). Flight plans were designed to track upwind and downwind to allow simple box modelling of the CH_4 flux. Flying height was at minimum safe altitude (typically 1000-1500 ft agl) with excursions to collect vertical profile information to 5000 ft agl. In-flight alterations were made to investigate interesting wetland features as seen during each sortie. Ground campaigns collected air samples adjacent to and downwind of wetlands located near Trinidad, Bolivia (14°49′S 64°54′W), with Tedlar sample bags filled at a range of locations and types. Tedlar bag samples were analysed post-campaign for CH_4 mixing ratio and $\delta^{13}C_{CH4}$ at Royal Holloway University of London (2).

Box-model fluxes were calculated from the uGGA measurements using the principles outlined in Hiller et al., (3) using a simple boundary layer budget approach. The concept defines a flow of CH_4 into and out of an imaginary box with length defined by the flight track length parallel to the wind vector. This very simple model requires the assumption that the surface of the box is emitting homogeneously and consistently, allowing a CH_4 surface flux to be estimated from the gradient of CH_4 mixing ratio per unit length along the wind vector. The PBL was determined as an average for each flight using profiles of air temperature measured during profile ascents and descents on the aircraft. The assumption of constant PBL height (determined from averaged heights measured during in-flight profile climbs), and that exchange of air across the PBL is negligibly small, the calculated CH_4 gradients for each flight are 0.3 ppb km^{-1} and 2.1 ppb km^{-1}. Full details of this method is found in Hiller et al., (3).

The GEOS-Chem inverse modeling methodology followed a Bayesian synthesis inversion framework (4). The state vector included 100 elements, 99 corresponding to emissions and one describing the baseline mole fraction. Measurements were averaged into 1-minute means. Model-measurement uncertainties included the standard deviation of measurements within each one-minute period and a fixed 8 ppb model uncertainty. A flat prior emissions distribution was used within the Llanos de Moxos basin with emissions of 48 mg CH_4/m^2/day. A nested GEOS-Chem simulation at 0.25° x 0.3125° was used to map the relationship between emissions and aircraft measurements in a regional domain bounded by 24 to 0 °S and 75 to 55 °W. Initial boundary conditions for the nested domain were created by a global GEOS-Chem simulation at 2° x 2.5°. Outside of the Llanos de Moxos basin, prior emissions followed those used in Maasakkers et al., (5).

The NAME model inversion was carried out using footprints simulated from the NAME Lagrangian particle dispersion model and a hierarchical Bayesian Markov chain Monte Carlo (MCMC) framework. NAME simulates the "footprint" or sensitivity between atmospheric mole fractions and surface emissions (see Manning et al., (6)). In this application, the NAME domain spanned -61 to 22.4 °N and -91.3 to -24.7 °E with meteorological drivers at a spatial resolution of 0.14° × 0.09° and temporal resolution of 3 hourly. A footprint was simulated for each minute of aircraft sampling. A priori fields comprised a flat prior emissions distribution within the domain of 48 mg CH_4/m^2/day and a priori boundary conditions which were the 2010-2018 average mole fraction fields from the CAMS CH_4 flux inversion product v18r1 (https://ads.atmosphere.copernicus.eu/cdsapp#!/dataset/cams-global-greenhouse-gas-inversion?tab=overview). 100 emission elements, 4 boundary condition elements, and an estimate of model error were solved for in the hierarchical inversion. The emissions elements solved for in the inversion were formed by aggregating model grid cells at the original NAME resolution into larger regions using a quadtree algorithm. This resulted in higher resolution estimates near the aircraft sampling locations. The four boundary condition elements scaled the a priori boundary conditions on each horizontal edge of the NAME domain. Emissions and boundary condition parameters were governed by truncated normal probability distribution functions (PDFs), truncated at 0 to prevent negative solutions. The mean of the PDFs were centred on *a priori* values, with a standard deviation of 15 times the mean value for emissions and 2% (approximately 30 ppb) for boundary conditions. Model errors were sampled from a uniform distribution of 0 to 30 ppb and thus were estimated as part of the inversion. The inversion was solved using MCMC with a No-U-Turn sampler for emissions and boundary conditions and a slice sampler for the model error hyperparmeter. A full description of the hierarchical Bayesian method can be found in Ganesan et al., (7) and Say et al., (8). The differing

results of the three methods may reflect the different treatment of meteorology and PBL height. In the NAME analysis, there is a strong negative correlation between modelled PBL height and observed CH mole fraction above background for both flights. In the GEOS-Chem analysis, a similar relationship between modelled PBL height and observed CH_4 mole fraction is only present for Flight A. Given that PBL height during Flight A was higher than during Flight B, this may be one reason why smaller emission fluxes are derived using NAME compared with GEOS-Chem.

GOSAT column averaged dry-air mole fraction of CH_4 (XCH_4) data from 2010 to 2020 (inclusive) was retrieved using a proxy retrieval that is less affected by cloud coverage (9). The raw data were averaged into 2° x 2.5° bins. The XCH_4 enhancement over the Llanos de Moxos was examined by comparing the GOSAT averaged XCH_4 data to XCH_4 mole fractions simulated by the GEOS-Chem atmospheric chemistry transport model. The model was run with a horizontal resolution of 2° x 2.5° for an 11-year period between 2010 and 2020, driven by meteorology fields from the MERRA2 reanalysis. The global model was driven by emissions from EDGAR v4.3.2 (10), WetCHARTs (11) and GFED4s (12). The 3D model fields were sampled at the time and location of GOSAT retrievals and convolved with the GOSAT averaging kernels and averaged into 2° x 2.5° bins. The monthly mean model fields were scaled to match the zonal mean of GOSAT retrievals in each 2° latitude band to minimize errors in the model distribution and growth rate due to errors in the model input emissions or loss fields.

The model run also included a tracer representing emissions just from the Llanos De Moxos region which resulted in a mean estimated XCH_4 enhancement in the same region of 5.3 ± 0.3 ppb between Jan-Mar. The mean difference between GOSAT and model between Jan-Mar was 34 ± 9 ppb (over six times larger than predicted by the model). For 2019 specifically, observed - model was 51 +/-16 ppb compared to the 11-year mean of 34 ppb. The 2019 modelled Llanos de Moxos enhancement was 4.7 +/-0.1 ppb compared to an average of 5.3 ppb over all years.

The mean model emission fluxes from the Llanos de Moxos region were 33 mg CH_4 mg/m^2/day, implying emissions of ~ 210 ± 55 mg mg/m^2/day required to be consistent with the GOSAT data. For 2019 the GOSAT comparison suggests ~ 355 ±115 mg/m^2/day. This estimate does not take into account errors in emissions from surrounding regions that may affect the enhancement over the Llanos de Moxos. A full analysis using inverse modelling would be needed to more robustly quantify this emission rate from satellite data. Nevertheless, this comparison of modelled and observed satellite XCH_4 provides further evidence for significant wet season CH_4 fluxes from the Llanos de Moxos wetlands. The scaling for the daily wet-season flux assumes a constant flux of 168 mg/m^2/day (the lowest flux estimate from all methods and from the most representative flight of the region as a whole) and over the 70000 km^2 of the seasonal wetland, this leads to a 0.012 Tg/day estimate from the region. For the purposes of upscaling to annual fluxes, the seasonality of the CH_4 flux is assumed to follow the same temporal pattern as the closest region analysed during the whole Amazonian basin analysis in Basso et al., (13).

Supplementary References

1. J. L. France *et al.*, Facility level measurement of offshore oil and gas installations from a medium-sized airborne platform: Method development for quantification and source identification of methane emissions. *Atmos Meas Tech* **14**, 71-88 (2021).
2. R. Fisher, D. Lowry, O. Wilkin, S. Sriskantharajah, E. G. Nisbet, High-precision, automated stable isotope analysis of atmospheric methane and carbon dioxide using continuous-flow isotope-ratio mass spectrometry. *Rapid Communications in Mass Spectrometry* **20**, 200-208 (2006).
3. R. V. Hiller *et al.*, Aircraft-based CH4 flux estimates for validation of emissions from an agriculturally dominated area in Switzerland. *Journal of Geophysical Research: Atmospheres* **119**, 4874-4887 (2014).
4. I. G. Enting, *Inverse Problems in Atmospheric Constituent Transport*, Cambridge Atmospheric and Space Science Series (Cambridge University Press, Cambridge, 2002), DOI: 10.1017/CBO9780511535741.
5. J. D. Maasakkers *et al.*, Global distribution of methane emissions, emission trends, and OH concentrations and trends inferred from an inversion of GOSAT satellite data for 2010–2015. *Atmos. Chem. Phys.* **19**, 7859-7881 (2019).

6. A. J. Manning, S. O'Doherty, A. R. Jones, P. G. Simmonds, R. G. Derwent, Estimating UK methane and nitrous oxide emissions from 1990 to 2007 using an inversion modeling approach. *Journal of Geophysical Research: Atmospheres* **116** (2011).
7. A. L. Ganesan *et al.*, Characterization of uncertainties in atmospheric trace gas inversions using hierarchical Bayesian methods. *Atmos. Chem. Phys.* **14**, 3855-3864 (2014).
8. D. Say *et al.*, Emissions and Marine Boundary Layer Concentrations of Unregulated Chlorocarbons Measured at Cape Point, South Africa. *Environmental Science & Technology* **54**, 10514-10523 (2020).
9. R. J. Parker *et al.*, A decade of GOSAT Proxy satellite CH4 observations. *Earth Syst. Sci. Data* **12**, 3383-3412 (2020).
10. G. Janssens-Maenhout *et al.*, EDGAR v4.3.2 Global Atlas of the three major greenhouse gas emissions for the period 1970–2012. *Earth Syst. Sci. Data* **11**, 959-1002 (2019).
11. A. A. Bloom *et al.*, A global wetland methane emissions and uncertainty dataset for atmospheric chemical transport models (WetCHARTs version 1.0). *Geosci. Model Dev.* **10**, 2141-2156 (2017).
12. G. R. van der Werf *et al.*, Global fire emissions estimates during 1997–2016. *Earth Syst. Sci. Data* **9**, 697-720 (2017).
13. L. S. Basso *et al.*, Amazon methane budget derived from multi-year airborne observations highlights regional variations in emissions. *Communications Earth & Environment* **2**, 246 (2021).
